# 1-(3,5-Di­nitro­benzo­yl)-4-(2-meth­oxy­phen­yl)piper­azine

**DOI:** 10.1107/S2414314620015230

**Published:** 2020-11-20

**Authors:** Chayanna Harish Chinthal, Channappa N. Kavitha, Hemmige S. Yathirajan, Sabine Foro, Christopher Glidewell

**Affiliations:** a Department of Studies in Chemistry, University of Mysore, Manasagangotri, Mysuru-570 006, India; b Department of Chemistry, Maharani’s Science College for Women, Mysuru-570 001, India; c Institute of Materials Science, Darmstadt University of Technology, Alarich-Weiss-Strasse 2, D-64287 Darmstadt, Germany; d School of Chemistry, University of St Andrews, St Andrews, Fife KY16 9ST, UK; Sunway University, Malaysia

**Keywords:** synthesis, crystal, mol­ecular conformation, π–π stacking

## Abstract

In the title compound, C_18_H_18_N_4_O_6_, the piperazine ring adopts a chair conformation, the amidic N atom is planar (sum of angles = 360°) and the non-amidic N atom is pyramidal (343°). There are no hydrogen bonds of any kind in the crystal, but the mol­ecules are linked by two independent π(nitro­benzene)⋯π(meth­oxy­benzene) stacking inter­actions to form π-stacked sheets with inter-centroid separations of 3.8444 (12) and 3.9197 (12) Å.

## Abstract 2

The mol­ecules of the title compound are linked into sheets by two π–π stacking inter­actions.

## Graphical abstract

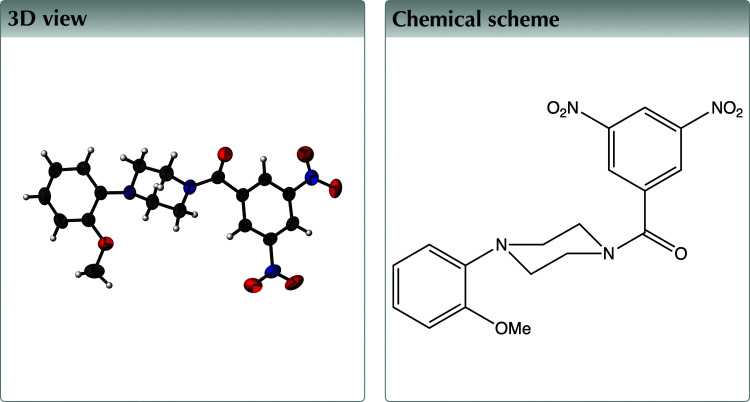

## Structure description

**Figure 1:**
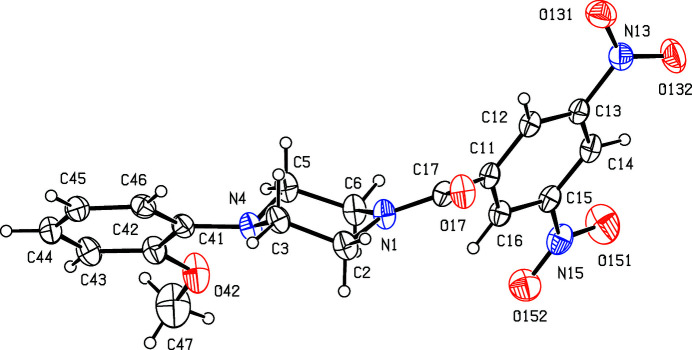
The mol­ecular structure of the title compound showing the atom-labelling scheme. Displacement ellipsoids are drawn at the 30% probability level.

Piperazines are found in a wide range of compounds that are active across a number of different therapeutic areas as they exhibit anti­bacterial, anti­depressant anti­fungal, anti­malarial, anti­psychotic, and anti­tumour activity (Brockunier *et al.*, 2004 ▸; Bogatcheva *et al.*, 2006 ▸), and a number of these areas have recently been reviewed (Elliott, 2011 ▸; Kharb *et al.*, 2012 ▸; Asif, 2015 ▸; Brito *et al.*, 2019 ▸). *N*-(2-Meth­oxy­phen­yl)piperazine has been used as a building block in the synthesis of both 5-HT_1 A_ receptor ligands (Orjales *et al.*, 1995 ▸) and dopamine D_2_ and D_3_ ligands (Hackling *et al.*, 2003 ▸), and also as a building block for the synthesis of derivatives exhibiting anti­depressant-like activity (Waszkielewicz *et al.*, 2015 ▸). The isomeric *N*-(4-meth­oxy­phen­yl)piperazine has been found to inhibit the re-uptake and accelerate the release of mono­amine neurotransmitters such as dopamine and serotonin, with a mechanism of action similar to that of recreational drugs such as amphetamines, but with significantly lower abuse potential (Nagai *et al.*, 2007 ▸). We have recently reported the structures of a range of 1-aroyl-4-(4-meth­oxy­phen­yl)piperazines (Harish Chinthal *et al.*, 2020 ▸), and in a continuation of that work, we report here the structure of the title compound (Fig. 1 ▸), which was prepared using a carbodi­imide-mediated condensation reaction between *N*-(2-meth­oxy­phen­yl)piperazine and 3,5-di­nitro­benzoic acid.

The piperazine ring in the title compound (Fig. 1 ▸) adopts a conformation that is close to an ideal chair form. The ring-puckering angle θ (Cremer & Pople, 1975 ▸), calculated for the atom sequence (N1,C2,C3,N4,C5,C6) is 12.69 (18)°, whereas this value would be zero for an ideal chair form (Boeyens, 1978 ▸). The geometry at the amidic atom N1 is planar within experimental uncertainty, but that at N4 is markedly pyramidal: the exocyclic substituents at both of these atoms occupy equatorial sites. In the di­nitro­benezene ring, the two nitro groups are both rotated out of the ring plane; the nitro groups bonded to atoms C13 and C15 make dihedral angles with the ring (C11–C16) of 20.52 (9) and 2.34 (12)°, respectively, with a dihedral angle of 22.09 (10)° between the planes of the two nitro groups, so that the rotations occur in a conrotatory sense. In the 2-meth­oxy­benzene substituent, the meth­oxy atom C47 is nearly coplanar with the adjacent ring, with a displacement from the ring plane of only 0.308 (5) Å. Associated with this near planarity, the two exocyclic angles at C42 are markedly different. Thus, C41—C42—O42 is 115.51 (16)° and C43—C42—O42 is 124.36 (18)°, as typically found in planar or near-planar alk­oxy­arenes (Seip & Seip, 1973 ▸; Ferguson *et al.*, 1996 ▸).

**Figure 2:**
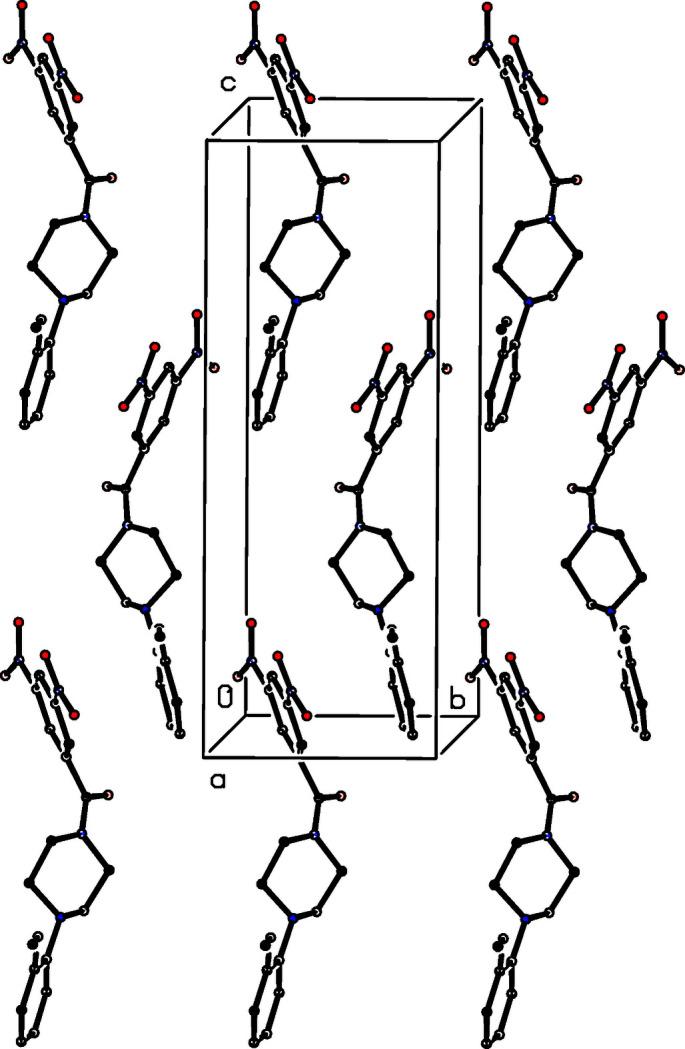
A view of the mol­ecular packing of the title compound showing the formation of a π-stacked sheet lying parallel to (100). For the sake of clarity, the H atoms have been omitted.

Despite the presence within the mol­ecule of six O atoms and two N atoms, all of which are potential hydrogen-bond acceptors, the structure contains no inter­molecular C—H⋯O or C—H⋯N hydrogen bonds, nor are there any C—H⋯π(arene) inter­actions. However, two π–π stacking inter­actions are present. The nitro­benzene ring at (*x*, *y*, *z*) makes a dihedral angle of 8.44 (9)° with the meth­oxy­benzene rings at both (*x*, 1 − *y*, 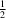 + *z*) and (*x*, 2 − *y*, 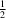 + *z*), *i.e*. in the mol­ecules related to the reference mol­ecule by the *c*-glide planes at *y* = 0.5 and 1, respectively. The ring-centroid separations are 3.9197 (12) and 3.8444 (12) Å, respectively, and the shortest distances between the centroid of one ring and the plane of the other are 3.3822 (8) and 3.2468 (8) Å, respectively, leading to the formation of a π-stacked sheet lying parallel to (100) in the domain 0.25 < *x* < 0.5 (Fig. 2 ▸). Three other sheets of this type pass through the unit cell, in the domains 0 < *x* < 0.25, 0.5 < *x* < 0.75, and 0.75 < *x* < 1.0, but there are no direction-specific inter­actions between adjacent sheets.

## Synthesis and crystallization

For the synthesis of the title compound, 1-(3-di­methyl­amino­prop­yl)-3-ethyl­carbodimide (134 mg, 0.7 mmol), 1-hy­droxy­benzotriazole (68 mg, 0.5 mmol) and tri­ethyl­amine (0.5 ml, 1.5 mmol) were added to a solution of 3,5-di­nitro­benzoic acid (114 mg, 0.5 mmol) in methanol (10 ml). This mixture was heated to 323 K, with stirring, for a few minutes before being set aside at ambient temperature. After two days, a solution of *N*-(2-meth­oxy­phen­yl)piperazine (100 mg, 0.52 mmol) in *N*,*N*-di­methyl­formamide (5 ml) was added and the resulting mixture was stirred overnight at ambient temperature. When the reaction was complete, as judged using thin layer chromatography, the mixture was quenched with water (10 ml) and extracted with ethyl acetate (20 ml). The organic fraction was separated and washed successively with an aqueous hydro­chloric acid solution (1 mol dm^−3^), a saturated solution of sodium hydrogen carbonate and finally with brine. The organic phase was dried over anhydrous sodium sulfate, the solvent was removed under reduced pressure, and the resulting product was recrystallized from methanol-ethyl acetate (1:1, *v*/*v*), m.p. 390–392 K. Crystals suitable for single-crystal X-ray diffraction were grown by slow evaporation, at ambient temperature and in the presence of air, of its ethyl acetate solution.

## Refinement

Crystal data, data collection and structure refinement details are summarized in Table 1 ▸.

## Supplementary Material

Crystal structure: contains datablock(s) global, I. DOI: 10.1107/S2414314620015230/tk4065sup1.cif

Structure factors: contains datablock(s) I. DOI: 10.1107/S2414314620015230/tk4065Isup2.hkl

Supporting information file. DOI: 10.1107/S2414314620015230/tk4065Isup3.cml

CCDC reference: 2044513

Additional supporting information:  crystallographic information; 3D view; checkCIF report

## Figures and Tables

**Table 1:** Experimental details

Crystal data	
Chemical formula	C_18_H_18_N_4_O_6_
*M*_r_	386.36
Crystal system, space group	Monoclinic, *C*2/*c*
Temperature (K)	296
*a*, *b*, *c* (Å)	25.348 (2), 7.3059 (5), 19.347 (1)
β (°)	94.190 (6)
*V* (Å^3^)	3573.3 (4)
*Z*	8
Radiation type	Mo *K*α
μ (mm^−1^)	0.11
Crystal size (mm)	0.46 × 0.32 × 0.22

Data collection	
Diffractometer	Oxford Diffraction Xcalibur diffractometer with Sapphire CCD detector
Absorption correction	Multi-scan (*CrysAlis RED*; Oxford Diffraction, 2009 ▸)
*T*_min_, *T*_max_	0.918, 0.976
No. of measured, independent and observed [*I* > 2σ(*I*)] reflections	7735, 3854, 2600
*R*_int_	0.016
(sin θ/λ)_max_ (Å^−1^)	0.657

Refinement	
*R*[*F* ^2^ > 2σ(*F* ^2^)], *wR*(*F* ^2^), *S*	0.047, 0.113, 1.02
No. of reflections	3854
No. of parameters	253
H-atom treatment	H-atom parameters constrained
Δρ_max_, Δρ_min_ (e Å^−3^)	0.22, −0.19

Computer programs: *CrysAlis CCD* (Oxford Diffraction, 2009 ▸), *CrysAlis RED* (Oxford Diffraction, 2009 ▸), *SHELXT* (Sheldrick, 2015*a* ▸), *SHELXL2014* (Sheldrick, 2015*b* ▸) and *PLATON* (Spek, 2020 ▸).

## full crystallographic data

### Crystal data

**Table:**

C_18_H_18_N_4_O_6_	*F*(000) = 1616
*M**_r_* = 386.36	*D*_x_ = 1.436 Mg m^−^^3^
Monoclinic, *C*2/*c*	Mo *K*α radiation, λ = 0.71073 Å
*a* = 25.348 (2) Å	Cell parameters from 3854 reflections
*b* = 7.3059 (5) Å	θ = 2.6–27.1°
*c* = 19.347 (1) Å	µ = 0.11 mm^−^^1^
β = 94.190 (6)°	*T* = 296 K
*V* = 3573.3 (4) Å^3^	Block, yellow
*Z* = 8	0.46 × 0.32 × 0.22 mm

### Data collection

**Table:**

Oxford Diffraction Xcalibur diffractometer with Sapphire CCD detector	3854 independent reflections
Radiation source: Enhance (Mo) X-ray Source	2600 reflections with *I* > 2σ(*I*)
Graphite monochromator	*R*_int_ = 0.016
ω scans	θ_max_ = 27.9°, θ_min_ = 2.6°
Absorption correction: multi-scan (*CrysAlis RED*; Oxford Diffraction, 2009)	*h* = −33→19
*T*_min_ = 0.918, *T*_max_ = 0.976	*k* = −7→9
7735 measured reflections	*l* = −25→22

### Refinement

**Table:**

Refinement on *F*^2^	Primary atom site location: difference Fourier map
Least-squares matrix: full	Hydrogen site location: inferred from neighbouring sites
*R*[*F*^2^ > 2σ(*F*^2^)] = 0.047	H-atom parameters constrained
*wR*(*F*^2^) = 0.113	*w* = 1/[σ^2^(*F*_o_^2^) + (0.0413*P*)^2^ + 2.4765*P*] where *P* = (*F*_o_^2^ + 2*F*_c_^2^)/3
*S* = 1.02	(Δ/σ)_max_ < 0.001
3854 reflections	Δρ_max_ = 0.22 e Å^−^^3^
253 parameters	Δρ_min_ = −0.19 e Å^−^^3^
0 restraints	

### Special details

**Table:**

Geometry. All esds (except the esd in the dihedral angle between two l.s. planes) are estimated using the full covariance matrix. The cell esds are taken into account individually in the estimation of esds in distances, angles and torsion angles; correlations between esds in cell parameters are only used when they are defined by crystal symmetry. An approximate (isotropic) treatment of cell esds is used for estimating esds involving l.s. planes.

### Fractional atomic coordinates and isotropic or equivalent isotropic displacement parameters (Å^2^)

**Table:**

	*x*	*y*	*z*	*U*_iso_*/*U*_eq_	
N1	0.59240 (6)	0.5953 (2)	0.34788 (7)	0.0459 (4)	
C2	0.57693 (8)	0.4792 (3)	0.28802 (9)	0.0488 (5)	
H2A	0.5431	0.4226	0.2947	0.059*	
H2B	0.6028	0.3826	0.2847	0.059*	
C3	0.57283 (7)	0.5876 (3)	0.22156 (9)	0.0453 (5)	
H3A	0.5647	0.5066	0.1825	0.054*	
H3B	0.5447	0.6774	0.2226	0.054*	
N4	0.62303 (6)	0.6787 (2)	0.21428 (7)	0.0454 (4)	
C5	0.63231 (8)	0.8121 (3)	0.27028 (9)	0.0501 (5)	
H5A	0.6030	0.8978	0.2694	0.060*	
H5B	0.6643	0.8806	0.2636	0.060*	
C6	0.63767 (8)	0.7159 (3)	0.33925 (9)	0.0500 (5)	
H6A	0.6700	0.6442	0.3426	0.060*	
H6B	0.6401	0.8060	0.3762	0.060*	
C17	0.56572 (7)	0.5803 (3)	0.40509 (9)	0.0409 (4)	
O17	0.52443 (5)	0.4943 (2)	0.40619 (6)	0.0563 (4)	
C11	0.58807 (7)	0.6641 (2)	0.47192 (9)	0.0391 (4)	
C12	0.55244 (7)	0.7415 (2)	0.51449 (9)	0.0404 (4)	
H12	0.5171	0.7561	0.4988	0.048*	
C13	0.56989 (7)	0.7962 (2)	0.58008 (9)	0.0405 (4)	
C14	0.62151 (7)	0.7749 (3)	0.60631 (9)	0.0444 (5)	
H14	0.6325	0.8105	0.6512	0.053*	
C15	0.65608 (7)	0.6986 (3)	0.56286 (9)	0.0424 (4)	
C16	0.64053 (7)	0.6426 (3)	0.49661 (9)	0.0416 (4)	
H16	0.6649	0.5909	0.4687	0.050*	
C41	0.63414 (7)	0.7360 (2)	0.14693 (9)	0.0417 (4)	
C42	0.68700 (8)	0.7778 (3)	0.13438 (10)	0.0499 (5)	
C43	0.69945 (8)	0.8298 (3)	0.06918 (10)	0.0555 (5)	
H43	0.7342	0.8598	0.0614	0.067*	
C44	0.66072 (9)	0.8375 (3)	0.01523 (10)	0.0545 (5)	
H44	0.6694	0.8741	−0.0286	0.065*	
C45	0.60985 (8)	0.7918 (3)	0.02602 (10)	0.0506 (5)	
H45	0.5840	0.7940	−0.0107	0.061*	
C46	0.59664 (7)	0.7421 (3)	0.09169 (9)	0.0444 (5)	
H46	0.5618	0.7123	0.0986	0.053*	
O42	0.72307 (6)	0.7570 (3)	0.18968 (8)	0.0780 (5)	
C47	0.77628 (10)	0.7698 (5)	0.18064 (16)	0.1038 (11)	
H47A	0.7961	0.7520	0.2243	0.156*	
H47B	0.7859	0.6778	0.1485	0.156*	
H47C	0.7839	0.8888	0.1628	0.156*	
N13	0.53195 (7)	0.8793 (2)	0.62448 (9)	0.0510 (4)	
O131	0.49148 (6)	0.9456 (2)	0.59666 (8)	0.0682 (4)	
O132	0.54302 (7)	0.8798 (2)	0.68663 (7)	0.0744 (5)	
N15	0.71189 (7)	0.6755 (3)	0.58839 (9)	0.0554 (5)	
O151	0.72524 (7)	0.7321 (3)	0.64600 (9)	0.0911 (6)	
O152	0.74214 (6)	0.6030 (3)	0.55072 (8)	0.0734 (5)	

### Atomic displacement parameters (Å^2^)

**Table:**

	*U*^11^	*U*^22^	*U*^33^	*U*^12^	*U*^13^	*U*^23^
N1	0.0490 (9)	0.0525 (10)	0.0361 (8)	−0.0173 (8)	0.0028 (7)	−0.0040 (7)
C2	0.0518 (11)	0.0507 (12)	0.0442 (11)	−0.0153 (9)	0.0053 (9)	−0.0092 (9)
C3	0.0437 (10)	0.0535 (12)	0.0390 (10)	−0.0098 (9)	0.0039 (8)	−0.0086 (9)
N4	0.0449 (9)	0.0556 (10)	0.0362 (8)	−0.0124 (8)	0.0061 (7)	−0.0050 (7)
C5	0.0561 (12)	0.0549 (12)	0.0398 (10)	−0.0182 (10)	0.0063 (9)	−0.0046 (9)
C6	0.0510 (12)	0.0591 (13)	0.0398 (10)	−0.0185 (10)	0.0031 (9)	−0.0026 (9)
C17	0.0407 (10)	0.0440 (11)	0.0374 (10)	−0.0040 (9)	−0.0012 (8)	0.0053 (8)
O17	0.0485 (8)	0.0740 (10)	0.0465 (8)	−0.0219 (7)	0.0027 (6)	−0.0025 (7)
C11	0.0422 (10)	0.0406 (10)	0.0342 (9)	−0.0085 (8)	0.0009 (8)	0.0063 (8)
C12	0.0395 (10)	0.0431 (11)	0.0380 (10)	−0.0057 (8)	−0.0004 (8)	0.0081 (8)
C13	0.0458 (10)	0.0391 (10)	0.0367 (9)	−0.0053 (8)	0.0034 (8)	0.0069 (8)
C14	0.0522 (12)	0.0457 (11)	0.0343 (9)	−0.0115 (9)	−0.0039 (8)	0.0067 (8)
C15	0.0403 (10)	0.0447 (11)	0.0414 (10)	−0.0081 (8)	−0.0031 (8)	0.0097 (8)
C16	0.0407 (10)	0.0453 (11)	0.0388 (10)	−0.0050 (8)	0.0023 (8)	0.0053 (8)
C41	0.0453 (11)	0.0431 (11)	0.0373 (10)	−0.0001 (8)	0.0065 (8)	−0.0042 (8)
C42	0.0441 (11)	0.0623 (13)	0.0435 (11)	−0.0026 (10)	0.0044 (9)	−0.0015 (10)
C43	0.0494 (12)	0.0655 (14)	0.0535 (12)	−0.0043 (10)	0.0165 (10)	0.0001 (11)
C44	0.0712 (15)	0.0538 (13)	0.0398 (11)	0.0026 (11)	0.0129 (10)	0.0016 (9)
C45	0.0619 (13)	0.0500 (12)	0.0392 (10)	0.0062 (10)	−0.0012 (9)	−0.0038 (9)
C46	0.0442 (11)	0.0454 (11)	0.0435 (11)	0.0006 (9)	0.0024 (8)	−0.0077 (9)
O42	0.0442 (9)	0.1365 (16)	0.0528 (9)	−0.0127 (9)	0.0002 (7)	0.0093 (9)
C47	0.0485 (15)	0.172 (3)	0.090 (2)	−0.0117 (18)	−0.0041 (13)	0.014 (2)
N13	0.0578 (11)	0.0499 (10)	0.0455 (10)	−0.0049 (8)	0.0061 (8)	0.0001 (8)
O131	0.0630 (10)	0.0750 (11)	0.0665 (10)	0.0149 (9)	0.0039 (8)	−0.0055 (8)
O132	0.0859 (11)	0.0987 (13)	0.0391 (8)	0.0054 (10)	0.0084 (7)	−0.0087 (8)
N15	0.0471 (10)	0.0657 (12)	0.0513 (11)	−0.0066 (9)	−0.0102 (9)	0.0101 (9)
O151	0.0624 (11)	0.1437 (18)	0.0629 (11)	0.0005 (11)	−0.0254 (8)	−0.0137 (11)
O152	0.0475 (9)	0.0979 (13)	0.0744 (11)	0.0077 (9)	0.0014 (8)	0.0013 (10)

### Geometric parameters (Å, º)

**Table:**

N1—C17	1.343 (2)	C14—C15	1.376 (3)
N1—C2	1.466 (2)	C14—H14	0.9300
N1—C6	1.466 (2)	C15—C16	1.375 (2)
C2—C3	1.507 (3)	C15—N15	1.474 (2)
C2—H2A	0.9700	C16—H16	0.9300
C2—H2B	0.9700	C41—C46	1.378 (2)
C3—N4	1.452 (2)	C41—C42	1.412 (3)
C3—H3A	0.9700	C42—O42	1.364 (2)
C3—H3B	0.9700	C42—C43	1.376 (3)
N4—C41	1.416 (2)	C43—C44	1.381 (3)
N4—C5	1.463 (2)	C43—H43	0.9300
C5—C6	1.506 (3)	C44—C45	1.363 (3)
C5—H5A	0.9700	C44—H44	0.9300
C5—H5B	0.9700	C45—C46	1.386 (3)
C6—H6A	0.9700	C45—H45	0.9300
C6—H6B	0.9700	C46—H46	0.9300
C17—O17	1.222 (2)	O42—C47	1.376 (3)
C17—C11	1.504 (2)	C47—H47A	0.9600
C11—C12	1.386 (2)	C47—H47B	0.9600
C11—C16	1.389 (2)	C47—H47C	0.9600
C12—C13	1.373 (2)	N13—O132	1.215 (2)
C12—H12	0.9300	N13—O131	1.223 (2)
C13—C14	1.377 (3)	N15—O151	1.213 (2)
C13—N13	1.467 (2)	N15—O152	1.217 (2)
			
C17—N1—C2	118.83 (15)	C14—C13—N13	118.48 (16)
C17—N1—C6	126.05 (15)	C15—C14—C13	116.80 (17)
C2—N1—C6	115.10 (14)	C15—C14—H14	121.6
N1—C2—C3	111.60 (16)	C13—C14—H14	121.6
N1—C2—H2A	109.3	C16—C15—C14	122.55 (17)
C3—C2—H2A	109.3	C16—C15—N15	118.76 (18)
N1—C2—H2B	109.3	C14—C15—N15	118.70 (17)
C3—C2—H2B	109.3	C15—C16—C11	119.27 (17)
H2A—C2—H2B	108.0	C15—C16—H16	120.4
N4—C3—C2	108.50 (15)	C11—C16—H16	120.4
N4—C3—H3A	110.0	C46—C41—C42	117.95 (17)
C2—C3—H3A	110.0	C46—C41—N4	123.42 (16)
N4—C3—H3B	110.0	C42—C41—N4	118.50 (16)
C2—C3—H3B	110.0	O42—C42—C43	124.36 (18)
H3A—C3—H3B	108.4	O42—C42—C41	115.51 (16)
C41—N4—C3	117.34 (14)	C43—C42—C41	120.09 (18)
C41—N4—C5	116.91 (15)	C42—C43—C44	120.42 (19)
C3—N4—C5	109.17 (14)	C42—C43—H43	119.8
N4—C5—C6	110.14 (16)	C44—C43—H43	119.8
N4—C5—H5A	109.6	C45—C44—C43	120.17 (18)
C6—C5—H5A	109.6	C45—C44—H44	119.9
N4—C5—H5B	109.6	C43—C44—H44	119.9
C6—C5—H5B	109.6	C44—C45—C46	119.96 (19)
H5A—C5—H5B	108.1	C44—C45—H45	120.0
N1—C6—C5	111.15 (15)	C46—C45—H45	120.0
N1—C6—H6A	109.4	C41—C46—C45	121.36 (18)
C5—C6—H6A	109.4	C41—C46—H46	119.3
N1—C6—H6B	109.4	C45—C46—H46	119.3
C5—C6—H6B	109.4	C42—O42—C47	119.98 (18)
H6A—C6—H6B	108.0	O42—C47—H47A	109.5
O17—C17—N1	122.66 (17)	O42—C47—H47B	109.5
O17—C17—C11	117.63 (16)	H47A—C47—H47B	109.5
N1—C17—C11	119.65 (16)	O42—C47—H47C	109.5
C12—C11—C16	119.39 (16)	H47A—C47—H47C	109.5
C12—C11—C17	117.14 (16)	H47B—C47—H47C	109.5
C16—C11—C17	122.74 (17)	O132—N13—O131	124.05 (18)
C13—C12—C11	119.23 (17)	O132—N13—C13	117.84 (17)
C13—C12—H12	120.4	O131—N13—C13	118.10 (16)
C11—C12—H12	120.4	O151—N15—O152	123.56 (18)
C12—C13—C14	122.75 (18)	O151—N15—C15	117.73 (19)
C12—C13—N13	118.77 (17)	O152—N15—C15	118.71 (17)
			
C17—N1—C2—C3	133.54 (18)	C12—C11—C16—C15	0.0 (3)
C6—N1—C2—C3	−48.0 (2)	C17—C11—C16—C15	−169.91 (17)
N1—C2—C3—N4	56.1 (2)	C3—N4—C41—C46	13.0 (3)
C2—C3—N4—C41	159.37 (16)	C5—N4—C41—C46	−119.6 (2)
C2—C3—N4—C5	−64.7 (2)	C3—N4—C41—C42	−162.80 (17)
C41—N4—C5—C6	−159.92 (16)	C5—N4—C41—C42	64.6 (2)
C3—N4—C5—C6	63.9 (2)	C46—C41—C42—O42	−175.49 (18)
C17—N1—C6—C5	−135.51 (19)	N4—C41—C42—O42	0.5 (3)
C2—N1—C6—C5	46.2 (2)	C46—C41—C42—C43	2.5 (3)
N4—C5—C6—N1	−53.2 (2)	N4—C41—C42—C43	178.50 (18)
C2—N1—C17—O17	−11.0 (3)	O42—C42—C43—C44	176.3 (2)
C6—N1—C17—O17	170.74 (19)	C41—C42—C43—C44	−1.4 (3)
C2—N1—C17—C11	166.07 (17)	C42—C43—C44—C45	−0.7 (3)
C6—N1—C17—C11	−12.2 (3)	C43—C44—C45—C46	1.7 (3)
O17—C17—C11—C12	−39.5 (2)	C42—C41—C46—C45	−1.5 (3)
N1—C17—C11—C12	143.26 (18)	N4—C41—C46—C45	−177.28 (17)
O17—C17—C11—C16	130.56 (19)	C44—C45—C46—C41	−0.6 (3)
N1—C17—C11—C16	−46.7 (3)	C43—C42—O42—C47	−6.7 (4)
C16—C11—C12—C13	0.4 (3)	C41—C42—O42—C47	171.1 (2)
C17—C11—C12—C13	170.85 (16)	C12—C13—N13—O132	159.68 (18)
C11—C12—C13—C14	−1.1 (3)	C14—C13—N13—O132	−19.7 (3)
C11—C12—C13—N13	179.48 (15)	C12—C13—N13—O131	−21.2 (3)
C12—C13—C14—C15	1.4 (3)	C14—C13—N13—O131	159.43 (18)
N13—C13—C14—C15	−179.23 (16)	C16—C15—N15—O151	177.25 (19)
C13—C14—C15—C16	−1.0 (3)	C14—C15—N15—O151	−2.9 (3)
C13—C14—C15—N15	179.17 (16)	C16—C15—N15—O152	−2.0 (3)
C14—C15—C16—C11	0.3 (3)	C14—C15—N15—O152	177.83 (18)
N15—C15—C16—C11	−179.80 (16)		
